# GEOGRAPHIC PROXIMITY TO NEIGHBORHOOD RESOURCES AND DEPRESSIVE SYMPTOMS AMONG KOREAN OLDER ADULTS

**DOI:** 10.1093/geroni/igac059.1996

**Published:** 2022-12-20

**Authors:** JuHyeong Lee, Donghee Shin, Yeoju Kim, Giyeon Kim

**Affiliations:** Chung-Ang University, Seoul, Seoul-t'ukpyolsi, Republic of Korea; Chung-Ang University, Seoul, Seoul-t'ukpyolsi, Republic of Korea; Chung-Ang University, Seoul, Seoul-t'ukpyolsi, Republic of Korea; Chung-Ang University, Seoul, Seoul-t'ukpyolsi, Republic of Korea

## Abstract

Given the importance of geographic proximity to neighborhood resources especially during the COVID-19 pandemic, this study examine whether the relationship between geographic proximity to neighborhood resources (e.g. hospitals, public transportation, etc.) and depressive symptoms varied by geographic location (i.e., rural vs. urban areas) among older adults in South Korea and whether this relationship was mediated by participation in social activities (e.g. education, club, community, etc.). The nationally representative samples, Korean older adults aged 65 or older, were drawn from the 2020 Survey of Living Conditions and Welfare Needs of Korean Older Persons (N=9,732, Urban=6,975, Rural=2,757). Hierarchical regression models, Baron and Kenny’s steps, and Sobel Test for the mediation effect were conducted. Results showed that geographic proximity was negatively associated with depressive symptoms in urban areas (B=-.041, p<.001), while positively associated in rural areas (B=.034, p<.01). Participation in social activities partially mediated the relationship in urban areas (Z=-2.162, p<.05), while there was no significant mediation effect in rural areas. Additionally, geographic proximity to hospitals or public transportation was significantly associated with depressive symptoms in rural areas. The findings suggest that geographic proximity to neighborhood resources helps older adults reduce social isolation, which may improve mental health of older adults living in urban areas during the pandemic. However, geographic proximity to neighborhood resources could make older adults living in rural areas become depressed, emphasizing that the characteristics of the urban and rural areas need to be considered to create an aged-friendly environment.

